# Effectiveness of Back care education Programme among school children: a systematic review of randomized controlled trials

**DOI:** 10.1186/s12887-024-04563-y

**Published:** 2024-02-02

**Authors:** Canice Chukwudi Anyachukwu, Confidence Chinemerem Amarah, Blessing Chiagozikam Atueyi, Ifeanyi Anthony, Martins Nweke, Ukachukwu Abaraogu

**Affiliations:** 1 Department of medical rehabilitation, Faculty of health sciences and technology, College of medicine, University of Nigeria, Enugu state, Nigeria; 2 Department of physiotherapy, Faculty of Health Sciences, University of Pretoria, Pretoria, South Africa; 3 Research Center for Health (ReaCH) Glasgow Caledonian University, Glasgow, UK; 4 Division of Biological Sciences and Health University of the West of Scotland, Lanarkshire, UK

**Keywords:** Back education, School children, School age, Back pain, Back care knowledge, Back school

## Abstract

**Study design:**

Systematic review of Randomised controlled trials.

**Objectives:**

With the increasing incidence of back pain among children and its untold implications to their future, back education tailored in an effective way would be indicated. However literature appears unsettled. This study aims to review available literature to determine the effect of school-based back education in preventing and managing low back pain in school children.

**Methods:**

Randomized controlled trials carried out on elementary and secondary school children of ages 6 to 18 years and published in English language were included. Back education taught in hospitals or other settings were excluded. Primary outcome was back pain prevalence and secondary outcomes were constituted from the study characteristics of selected studies which includes: back behavior, knowledge, postural habits, physical activity, fear-avoidance beliefs, back pack carriage, pain intensity, skills and self efficacy. Databases searched were PEDro, HINARI, PubMed, Cochrane, and Google Scholar. Available stiudies from 2000 to March 2022 were retrieved. Quality of studies were assessed using the PEDro scale. Obtained studies were descriptively analyzed.

**Results:**

A total 8420 studies were retrieved and 8 studies (with 1239 participants) were included in this review. Four studies each assessed back knowledge and back behavior, and two assessed back pain prevalence. There were improvements in back knowledge and back behaviour, but effectiveness of back care education on back pain prevalence was not conclusive.

Forms of education used involved the indirect method of conditioning the environment and the direct method which made use of theory, practical lessons and educational books and materials.

**Conclusion:**

Back care education programmes in schools are effective in improving back care knowledge, behavior and reduction in low back pain frequency. Reduction in back pain prevalence is not conclusive. Back care education could be incorporated as part of schools’ education programmes. Limitations include exclusion of non English language studies and inconsistent outcome measures.

**Funding source:**

None.

**Registration:**

This review protocol was registered under the International platform of Registered systematic review and meta-analysis protocol (INPLASY) with the registration number; INPLASY202310044 and DOI number; 10.37766/inplasy2023.1.0044

**Supplementary Information:**

The online version contains supplementary material available at 10.1186/s12887-024-04563-y.

## Introduction

Low back pain is increasingly prevalent among children and adolescents [1], as various studies have indicated its occurrence in the younger population [2–4]. In Nigeria specifically, adolescents exhibit a prevalence of approximately 25% for low back pain [5].

Common contributors to low back pain encompass various factors such as posture, underlying health conditions, improper lifting techniques, inadequate ergonomics, excessive weight and manner of carrying backpacks [4, 6]. Risk factors associated with back pain involve the weight of the backpack, the nature of school furniture, overall lifestyle, and a history of prior back pain experiences [7]. Additionally, an observed correlation exists between poor posture and the occurrence of low back pain in children [8]. Diverse approaches, both pharmacological and non-pharmacological, have been employed to mitigate back pain [9, 10]. Non-pharmacological strategies encompass a range of interventions including back care education, exercises, yoga, and acupuncture [11]. While back care education is promoted as a method for managing back pain, ongoing discussions persist about its effectiveness [12].

Back care education is an approach to prevent and maintain a healthy and pain free back [13]. The original Swedish Back School, established by Zachrisson-Forsell in 1969, aimed to diminish back pain by educating patients on proper back care [14]. This program spanned four lessons across a two-week period, each lasting about 15 minutes. The initial session covered back anatomy, the prevalence of back pain, and diverse treatment methods. Subsequent lessons addressed body biomechanics, the role of back muscles, posture, specific relaxation exercises, proper weight lifting techniques, and physical exercises [15]. Presently, the content and duration of back school programs exhibit substantial variation, yet their fundamental purpose remains unchanged [3, 16–18]. Integrating back care education into school curricula offers potential to enhance students’ understanding and implementation of back care practices [19, 20], which has proven pivotal in reducing back pain incidence within educational settings [21, 22]. Despite numerous studies on back care education [3, 16, 17, 23], there is a crucial need to consolidate existing evidence regarding the effectiveness of these school-based programs.

Thus, the aim of the review is to comprehensively examine various back care education programs implemented in schools targeting the prevention and management of low back pain among children and adolescents 18 years or younger, providing detailed insights into their effectiveness on specific outcomes.

## Methods

### Registration

This review was registered under the International platform of Registered systematic review and meta-analysis protocol (INPLASY) with the registration number; INPLASY202310044 and DOI number; 10.37766/inplasy2023.1.0044.

### Criteria for eligibility

This review included randomised controlled trials (RCTs) conducted in the elementary and secondary schools. Participants had to be students or pupils of the school aged between 6 to 18 years. Available studies on back care education programmes and back schools both as a preventive and management strategy for back pain carried out by teachers, physiotherapists or any other health care professionals was included.. The studies must have been published in English language.

However, studies on secondary data which such as systematic reviews, narrative reviews, scoping reviews were excluded and so were studies with no access to their full documents.

### Information sources and search

Search for information was performed using the following databases; PubMed, PEDro, Cochrane library, Google scholar and HINARI.

The search involved the use of medical subject headings, text terms, keywords and word variants that represented programmes on back care (back school OR back education programme OR back health OR postural education) and terms that captured their measure of effectiveness (efficacy OR effectiveness OR importance) in children (children OR adolescents OR school-aged children) combined using the Boolean operator “AND” (see Supplementary Document). The search applied no search limits during the search. These databases were searched from inception to 31/03/2022. Weekly alerts of new literature were received weekly until 16/12/2023.

### Study selection

Results of the search was exported to endnote and stored in Rayyan [24] from where duplicates were removed. Screening of studies was done independently by the primary reviewer. Articles selection and data extraction was performed by the primary reviewer, A.C.C and double-checked by a second reviewer, A.B.C.. Standardization of the procedure was required for regularity in method of data extraction used by the reviewers.

**Fig. 1:**
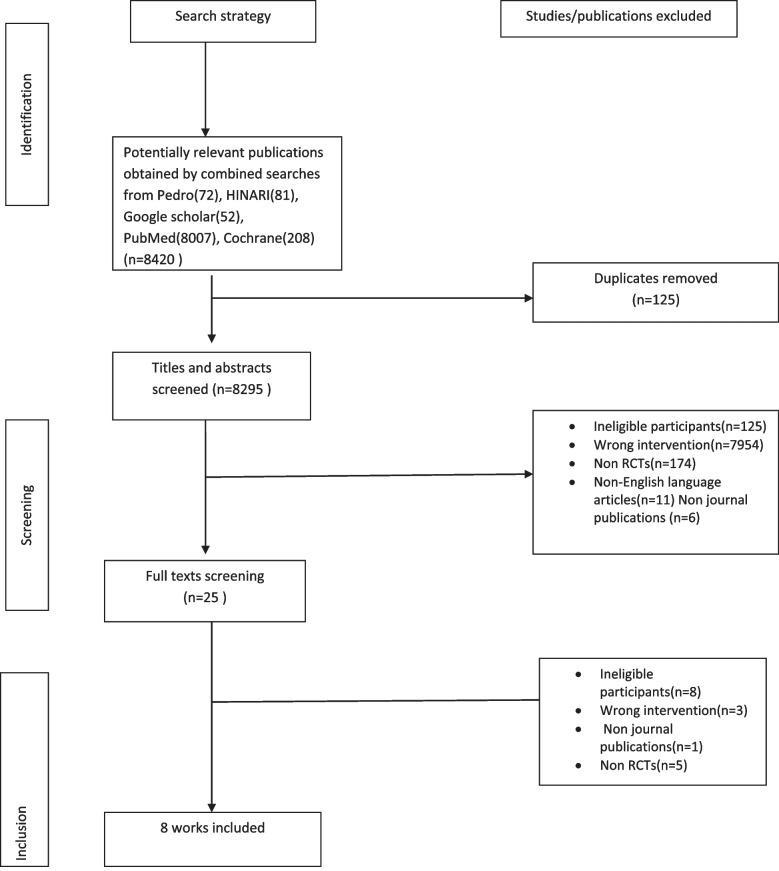
PRISMA flowchart

For each included trial, data was extracted regarding the participants (eligibility criteria), the interventions, the control and the outcome measures. Disagreements were resolved by discussion and reaching consensus or using a third reviewer, C.A.C. Details of the screening based on the eligibility criteria along with reasons for exclusion are presented in a flow chart (Fig. 1).

### Data collection

Data was collected by 2 individuals, A.C.C and A.B.C on the following variables: author’s study location, study characteristics (sample size, study design), participant’s characteristics (age, education level) which are recorded in an evidence table (Table 1). Other variables wherein data was synthesized include; intervention settings, the intervention accessor, period of the intervention, follow-up periods, study quality, outcomes accessed and their outcome measures.

**Table 1:** Data Extraction for included studies (I.G- Intervention group, C.G- Control group, LBP – low back pain)

S/ N	STUDY	COUNTRY	RESIDENTIAL FACILITY	PARTICIPANTS	INTERVENTION MODEL/CARE	CONTROL/COMPARISON	OUTCOME ACCESSED/OUTCOME MEASURE
1.	(Akbari-Chehrehbargh, Tavafian, & Montazeri, 2020) [25]	Tehran, Iran	Elementary schools	5th grade female school children. Sample size (104 pupils, IG = 52, CG = 52). Age (11 ± 1.0 years for both CG and IG)	Six sessions of T-bak educational program including four components: belief (one session), knowledge (one session), skills (two sessions) and self-efficacy (two sessions).Freq: once a week Dur: 1 hr	No therapy	Primary outcome; Improved back care related behavior (5-point Likert-type scale with total score ranging from 6 to 30). Secondary outcome; enhancement in beliefs (six-item scale), back care knowledge (multiple choice quiz), skills (checklist) and self-efficacy (four point Likert type scale).
2.	(Dullien, Grifka, & Jansen, 2018) [16]	Germany	Elementary school	176 pupils aged 10–12 years (mean age = 10.6 ± 0.44). CG = 86, IG = 90	Five lessons on back care, posture awareness training, back and abdominal muscle exercises	No therapy	Motor skills, back behavior, knowledge. Outcome measures; clinical orthopaedic exam, health questionnaire, motor test, back behaviour trial and knowledge test
3.	(Rodriguez-Garcia, Lopez-Minarro, & Santonja, 2013) [21]	Spain	Elementary and secondary schools	41 elementary school children (mean age 10.27 ± 0.31 years), 43 secondary school children (mean age 13.46 ± 0.68).sample size, *n* = 84, CG = 40, IG = 44	An organized physical education programme. Dur; 13mins Freq; 2 times a week	No therapy	Back pain frequency Pain intensity (visual analogue scale)
4.	(Habybabady R. H., et al., 2012) [18]	Iran	Elementary school	5th grade elementary schoolchildren. Sample size =404, CG = 201, 104 girls ad 97 boys. IG = 203, 101 girls and 102 boys	Education programme using educational pamphlets, Duration; 60 minutes.	No therapy	Knowledge and behavior (questionnaires)
5.	(Vidal-Conti & Galmes-Panades, 2022) [26]	Spain	Primary school	Schoolchildren aged 10–12 years. Sample size = 224. CG = 5 schools (*n* = 127), IG = 5 schools (*n* = 97).	Online training postural on class teachers, implementation of active breaks for classroom teachers, development of a postural education teaching unit, awareness of general school community.	No therapy	Prevalence of LBP (Self-administered questionnaires), daily postural habits {Back pain and body posture evaluation instrument (BackPEI)}
6.	(Vidal, et al., 2013) [27]	Spain	Primary schools	Primary school children aged 10–12 years AV = 10.7, SD = 0.672. sample size = 137,IG = 63, CG = 74	6 sessions of postural education program for 6 weeks(4 theoretical and 2 practical) Dur: 1 hr. Freq: once a week	Usual school curriculum	Try to load the minimum weight possible, school back pack carriage on both shoulders, belief that backpack do not affect the back, use of locker or something similar at school (questionnaires).
7.	(Cardon, de Clercq, Geldhof, Verstraete, &de Bourdeaudhuij, 2007) [28]	Belgium	Elementary schools	4th and 5th grade students. Mean age = 9.7 ± 0.7, range 8.1–12.0. sample size = 555, IG 1 = 190, 1G 2 = 193, CG = 172	IG 1 = Back care education programme consisting 6 lessons with 1 week interval. Physical activity promotion programme, 6 lessons at 1 wk. interval. IG 2 = Back care promotion condition	No therapy	back care behavior (observation), knowledge, fear-avoidance beliefs, back pain prevalence (questionnaire), and physical activity (accelerometer)
8.	(Kovacs, et al., 2011) [19]	Spain	Elementary schools	School children. Age = 8 years, sample size = 497, CG = 231, 1G = 266.	Comic book of the back	no intervention	Knowledge (questionnaires)

S/ N	Pre intervention measures	Post intervention measures	Pre control measures	Post control measures	RESULTS	CONCLUSION	QUALITY SCORE
1.	Behaviour (17.26 ± 4.97,*p* value- 0.36) Knowledge(4.16 ± 1.53, *p* value-0.65) Skills (13.26 ± 9.37, *p* value- 0.95) Self efficacy(10.66 ± 2.86, *p* value- 0.66) Beliefs (19.16 ± 4.19, *p* value- 0.24)	Behaviour (26.35 ± 3.61) Knowledge(4.30 ± 1.46) Skills(13.70 ± 10.18), Self efficacy(14.22 ± 2.17) Beliefs(26.31 ± 4.39)	Behaviour (18.30 ± 5.00) Knowledge(4.30 ± 1.46) Skills(13.70 ± 10.18) Self efficacy(10.2 ± 2.97) Beliefs(18.08 ± 4.83)	Behaviour (17.02 ± 5.59) Knowledge(4.16 ± 1.61) Skills(13.53 ± 10.18), Self efficacy(10.80 ± 2.73) Beliefs(18.18 ± 4.42)	There was a significant improvement on behaviour, beliefs, skills, self-efficacy and knowledge in the intervention group. No significant difference on outcomes assessed in the control group	T-bak educational program is effective in improving back care related behaviour among pupils	7
2.	Knowledge(14.42 ± 3.03, *p*- 0.001) behaviour (5.7 ± 1.9, *p* value- 0.005) motor skills (3.4 ± 3.8, *p* value< 0.001)	Knowledge(17.17 ± 2.84), behaviour (8.2 ± 2.0) motor skills (5.6 ± 3.9)	Knowledge(14.80 ± 5.05), behaviour (6.1 ± 1.7) motor skills (2.2 ± 3.0)	Knowledge(14.57 ± 4.42), behaviour (7.7 ± 2.1) motor skills (4.9 ± 4.0)	Improvement on back behaviour& knowledge among the IG. Increased Posture performance and improvement in spinal deformity seen in both IG and CG. no significant difference in back pain frequency & core muscle endurance in both groups	Teacher led back education programme should be included in schools.	6
3.	Pain frequency (9.5%)	Pain frequency 2.4%	Pain frequency 11.9%	Pain frequency 22.6%	Decrease in low back pain frequency in the IG and an increase in the CG	Children and adolescents subjected to the school physical education programme showed a reduction in low back pain frequency	8
4.	Knowledge (43.4 ± 12.93) Behaviour (53.3 ± 16.34)	Knowledge (74.5 ± 19.60) Behaviour (75.8 ± 18.58)	Knowledge (47.0 ± 12.76) Behaviour (54.7 ± 13.57)	Knowledge (48.1 ± 13.78) Behaviour (56.0 ± 16.43)	Significant increase in knowledge and behavior in the IG after one week and 3 months as compared to the CG.	Knowledge and behavior of children can be improved through educational programmes. It should be included in schools’ curriculum to ensure its sustainability	6
5.	Postural habits (2.86 ± 1.000) Last week prevalence (17.4%)	Postural habits (2.56 ± 1.108) Last week prevalence(15.5%)	Postural habits (2.93 ± 1.142) Last week prevalence (17.4%)	Postural habits (2.64 ± 1.067) Last week prevalence(18.6%)	No significant difference in low back pain prevalence and healthy postural habits both in CG ad IG	Postural education did not improve postural habits in children	5
6.	Last week LBP (19 ± 13.9%) Min wt (114 ± 83.2) Belief (19 ± 13.9) Carry backpack (121 ± 88.3)	Last week LBP (6 ± 9.5%) Min wt (48 ± 76.2) Belief (5 ± 7.9) Carry backpack (54 ± 85.7)	Last week LBP (19 ± 13.9%) Min wt (114 ± 83.2) Belief (19 ± 13.9) Carry backpack (121 ± 88.3)	Last week LBP (13 ± 17.6%) Min wt (66 ± 89.2) Belief (14 ± 18.9) Carry backpack (121 ± 88.3)	Repeated ANCOVA shows a significant increase in healthy backpack use in the IG	Children are able to learn healthy backpack habits which could prevent future LBP.	5
7.	Knowledge (1.0 ± 3.9) Behaviour (17.36 ± 4.82)	Knowledge (5.1 ± 2.9) Behaviour (25.44 ± 4.66)	Knowledge(0.7 ± 3.4) Behaviour (16.46 ± 4.20)	Knowledge (2.7 ± 3.0) Behaviour (18.48 ± 5.43)	Significant increase in back care behavior in both intervention groups than the control group Increase in fear avoidance in the control group different from the intervention groups. Increase in physical activity in the back care + physical activity promotion group.	It is important to incorporate back care education in the training of teachers. It should be also be integrated into the school curriculum	8
8.	Total median score; 8 (*p* value < 0.001)	9	7	9	Slight increase in knowledge in the IG	Small but valuable effects of the comic book of the back in improving children’s knowledge of appropriate methods for preventing and managing LBP	8

Data extraction for included studies

### Quality appraisal

The methodological quality of the included studies and the risk of bias was assessed independently using the PEDro scale. The PEDro scale is an effective tool for the measurement of the methodological feature of clinical trials [29–31]. The PEDro scale includes an 11 item which comprises external validity (item 1), internal validity (item 2–9) and statistical reporting (item 10–11). It relays internal validity and interpretability which is used to access each of the selected studies [30]. Quality is accessed by the level of score allocated. Scores less than 5 indicates low quality while scores greater than 5 indicates a high quality. Based on the PEDro assessment and sample size used, the level of evidence was assigned to each study. High quality RCTs (rated as high or excellent by PEDro with sample size ≥100) was considered as having level 1 evidence, whereas lower-quality RCTs (rated as low by PEDro with sample size less < 100) was considered level 2 evidence.

The scale were demarcated as Yes or No. A score of one was allocated to each “Yes” answer and zero to “No” answer. The overall score was reported as a tally of all yes answers out of 11 based on the appropriate answers for each study (see Table 2).

**Table 2:** Pedro scale for quality appraisal

s/n	STUDIES	Q1	Q2	Q3	Q4	Q5	Q6	Q7	Q8	Q9	Q10	Q11	TOTAL
1	(Akbari-Chehrehbargh, Tavafian, & Montazeri, 2020) [25]	1	1	1	1	0	0	0	1	1	1	1	7
2	(Dullien, Grifka, & Jansen, 2018) [16]	0	1	0	1	0	0	0	1	1	1	1	6
3	(Rodriguez-Garcia, Lopez-Minarro, & Santonja, 2013) [21]	1	1	1	1	1	1	0	1	1	1	0	8
4	(Habybabady R. H., et al., 2012) [18]	0	1	0	1	0	0	0	1	1	1	1	6
5	(Vidal-Conti & Galmes-Panades, 2022) [26]	1	1	0	1	0	0	0	1	0	1	1	5
6	(Vidal, et al., 2013) [27]	0	1	0	1	0	0	0	1	0	1	1	5
7	(Cardon, de Clercq, Geldhof, Verstraete, & de Bourdeaudhuij, 2007) [28]	0	1	0	1	1	1	1	1	1	1	0	8
8	(Kovacs, et al., 2011) [19]	1	1	1	1	1	0	1	1	1	1	0	8

#### Data synthesis

Data was synthesized separately to answer the objective questions. QualitativeSynthesis was used to analyze the extracted data. The main variable was the effect of back education on back pain prevalence and other outcomes and back education methods. Data was grouped based on the country the study was carried out, the type of professionals that carried out the interventions, sample size, age group of participants, outcomes and outcome measures (see Table 1).

## Results

Our search identified 8420 articles from five databases using the search strategies (see Supplementary Document). A hundred and 25 articles were excluded after deduplication. Total number of 8270 articles were excluded based on abstract and title screening and 17 works excluded were based on full text screening. Eight articles [32] were eligible for this systematic review. Details are presented in the PRISMA flowchart (Fig. 1).

### Quality appraisal

All of the studies had evidence of randomization, had their groups similar at baseline and had less than 15% dropout rate (100%). Most of the works certified the intention to treat analysis (75%) and 50% specified their eligibility criteria. However, few studies used an adequate blinding method (37.5%). 75% of the studies were of good quality (Pedro scale 6–8). Based on sample size and study quality, 62.5% of the included studies were of level 1 evidence while 37.5 were of level 2 evidence.

Studies that utilized non-practical aspect of learning were good quality trials and had level 1 evidence but just few studies that utilized practical lessons were of good quality (see Table 3).

**Table 3:** Summaryof methodological qualities of studies

METHODOLOGICAL QUALITY	NO OF STUDIES	PERCENTAGES
**PEDRO SCALE**		
Eligibility criteria	4	50%
Radom allocation	8	100%
Concealed allocation	3	37.5%
Groups similar at baseline	8	100%
Subject blinding	3	37.5%
Therapist blinding	1	12.5%
Assessor blinding	2	25%
Less than 15% dropouts	8	100%
Intention to treat analysis	6	75%
Between groups statistical comparisons	8	100%
Point measure and variability data	4	50%
**PEDRO TOTAL SCORE**		
Excellent quality (9, 10)	0	0%
Good quality (6–8)	6	75%
Fair quality (4, 5)	2	25%
Poor quality(0–4)	0	0%
**SAMPLE SIZE**		
≤100	1	12.5
≥100	7	87.5%
**LEVEL OF EVIDENCE**		
Level 1	5	62.5%
Level 2	3	37.5%

### Distribution of studies based on location and regional economic classification

Studies in this review were done in Iran (25%), Germany (12.5%), Spain (50%) and Belgium (12.5%). The World Bank classified countries based on their gross national income into low income, lower middle, upper middle income and high income countries [33]. 75% of the studies were done in Germany, Spain and Belgium which are high income countries. Only two of the studies were done in Iran, a low middle income country. Studies that utilized practical lessons were all done in high income countries.

### Outcomes assessed and their outcome measures

Most of the studies assessed back care behaviour (50%) and knowledge of back care (50%). Studies that assessed back behaviour, all used different measures (observation, 5-point scale, a back behaviour trial and questionnaires) while studies that assessed knowledge all made use of questionnaires. Other outcomes assessed include back care belief, skills, self-efficacy, back pain prevalence, pain intensity etc. (see Table 4).

**Table 4:** Outcomes and outcome measures

S/N	OUTCOMES	OUTCOME MEASURES	FREQUENCY	%
1.	Pain intensity	Visual analogue scale (VAS)	1	12.5%
2.	Knowledge	Questionnaires	4	50%
3.	Back care behavior	5point scale, questionnaires, observation	4	50%
4.	Back pain prevalence	Questionnaires	2	25%
5.	Postural habits	Back pain and body posture evaluation instrument	1	12.5%
6.	Back pack carriage	Questionnaires	1	12.5%
7.	Physical activity	Accelerometer	1	12.5%
8.	Fear-avoidance	Questionnaires	1	12.5%
9.	Beliefs	6point scale	1	12.5%
10.	Use of locker or alternate means	Questionnaires	1	12.5%
11.	Minimum weight loading of back packs	Questionnaires	1	12.5%
12.	Skills	Checklist	1	12.5%
13.	Self-efficacy	4point scale	1	12.5%

## Studies’ characteristics

### Type/method of education programme

Only 25% of the studies made the use of readable materials for educating the children [18, 19]. In one of the studies, an indirect form of learning was used in which teachers were trained and the general school community were educated through awareness [26]. Two of the studies (25%) had an education on a section of back care; good posture and correct use of back packs [26, 27].

### Studies’ settings

The studies were done in schools, elementary/primary schools. However, one of the studies included both primary and secondary schools [21].

### Individuals that assessed the outcomes

Teachers were the principal assessors in three of the included studies [16, 19, 21]. In the other studies, professionals like physical education instructor and health educator [25], sports scientists [26], occupational health experts [19] and physiotherapists [28] were the principal assessors of the outcome(s). In one study however, one of the researchers was the outcome assessor [27].

## Effects of back care education

### Effects of back care education on back behaviour

Back behavior was assessed in four studies with a total number of 1239 participants. In all the studies there was significant increase in back care behavior. Most of the works used lessons and practical sessions. Only one of the work made use of educational pamphlets [18].

### Effects of back care education on knowledge of back care

Knowledge was assessed in four studies with 1181 participants. Significant improvement of back care knowledge was noted in all the studies. One of the studies however reported a slight improvement [19]. Two of the studies [18, 19] used made use of educational reading materials (comic book and pamphlets) while the other two made use of lessons [16, 28].

### Effects of back care education on back pain intensity

According to one of the studies [19] that assessed back pain intensity, there was decreased frequency of low back pain in the intervention group. This study made use of an organized physical education programme.

### Effects of back care education on back pain prevalence

Two studies assessed the back pain prevalence. In one of the studies [28] there was a significant reduction in back pain prevalence, while the other study [26] showed no significant difference in back pain prevalence. This other study however, used an indirect method of educating the children.

### Effects of back care education on other outcomes

In the study that assessed postural habits, results show no significant improvement in postural habits [26] while studies that assessed back pack carriage [27], physical activity and back care beliefs [28], use of alternative measures of managing school load and minimum back pack loading [27], back skills and self-efficacy [25] all reported improvements in these outcomes.

## Discussion

The objective of this study was to ascertain the effectiveness of back care education programmes by reviewing available RCTs. Results shows that back care education programmes are effective in improving knowledge, behaviour, back pain intensity. Effectiveness on back care on back pain prevalence cannot be concluded as 50% of the studies that accessed back pain prevalence showed no specific difference.

### Method of education

Method of education used influences back behavior. Children could be described as activity loving, therefore an education type that is full of activity could be more of interest to them [8]. Several other studies [20, 23, 34] that used an activity-loving form of education also show improved outcomes. Back behaviour is important in bringing about low back pain reduction as it becomes the part of life of the children [35].

The use of educational pamphlet also brought about an increase in back behavior, this may be since the pupils’ source for this information from these reading materials themselves. Therefore, the information is likely to remain with them as they had to pay the price of getting it. Reading also widens imaginations and creativity. For children and teenagers, reading method of learning would even help the children devise measures towards their back care. This is beneficial as it is the pupils bringing up the methods themselves and not being imposed on them by their teachers or instructor. Hence there are high chances of following up with the learnt behavior. This reading method could also help in many other aspects of child development; vocabulary learning and improvement in levels of concentration [36]. On the other hand, this type of intervention could be less effective in that it is less interesting and exciting to the pupils. Reading also requires quite a lot of concentration which a child may not have developed [37].

### Outcomes

One of the studies [26], recorded no improvement in posture after back care education was carried out for a duration of four and half months, this can be attributed to the indirect method of education whereby the teachers were taught, and the environment was conditioned towards good posture adaptation. This method of education programme would likely not get the children to understand clearly the information being passed unto them as the main point is not explained to them. This method however proved different and can be beneficial as the whole school community was educated and the environment fashioned to encourage good posture [38].

However, a study by Kovacs, et al., [19] found out that education improved the children’s knowledge on low back pain prevention and management but the improvement was sure for a period of 3 months after the intervention. This intervention was carried out for a period of 3 months which is a good time to develop good skills with the knowledge gained. Family and cultural lifestyle could also affect how the children would easily adapt and continue in the already learned behaviors [39]. For back care education to remain effective, they must be a reinforcement of learned behaviour at home from by parents and guardians [40].

Education of the pupils also improved their knowledge. This gained knowledge then leads to behaviour. This may explain why most studies assessed both back knowledge and behaviour [16, 18, 25]. Knowledge can be defined as body of facts gained from education or experience [41], while education can be described as an enlightening experience [42]. Knowledge can therefore be said to be a good consequence of education [32]. Though knowledge alone cannot bring about behavioural change, it ensures that individuals know the need of a behaviour. Knowledge also presents the way to go about a thing which would make the development of the behaviour more possible to the individual [32]. Therefore back care education would equip students with the knowledge of back care, its benefits and consequences and how to adopt the behaviour; hence giving them a reason to inculcate the behaviour in their daily life [12].

On back pain frequency, there was no reduction in one of the studies [16]. Other factors causing back pain which were not accessed may contribute to the nil noticeable reduction seen. In a study by Solomou, Kraniotis, Rigopoulou, & Petsas [43], it was opined that underlying conditions like disc degenerative changes, Scheuermann’s disease [44], disc hernia and bulges contribute to increase in low back pain frequency. A study [45] also shows that osteoid osteoma can be a cause of low back pain.

### Study location

Based on geographical representation, most of the studies reviewed were done in high income countries. In a study [46], there were more dangers of inactivity in high income coutries compared to low income countries. In high income countries, there has been significant levels of inactivity owning to economic inequality [47]. Luxury and technological advancements can create room for sedentary lifestyle, obesity and increased screen time which are all risk factors for low back pain in children [48, 49]. Back education is therefore indicated in such countries. Back education programmes can also be seen in low- and middle-income countries due to cultural norms, harsh weather and inadequate sports facilities preventing physical activity and leading to the incidence of low back pain in these countries [47, 50].

### Intervention instructors

Education programme carried out in a school setting would ensure that they are monitored by the teachers; this would be effective as students spend quite many hours per week with their teachers. This would aid in reinforcing the desired behaviour. Other professionals like occupational health experts, physiotherapists, sports scientists may have the right knowledge, but teachers and parents can pass the information better to the children. Therefore, the need of educating the teachers and parents/guardians proves important [51].

## Conclusion

These education programmes include; theory and practical lessons and reading materials. These direct methods of education showed more effectiveness than indirect methods. Therefore they should be part of schools work as it would contribute to appropriate child development and health and combat future risks.

Back care education programmes in schools are effective in improving back care knowledge, behaviour, reducing pain intensity.

### Limitations

Meta analysis was not conducted.

The study however, did not assess the long term effects of back education.

### Supplementary Information

**Additional file 1.**

## Data Availability

Review data is available from the corresponding author on request.
