# Supplementary material for: Effectiveness of Back care education Programme among school children: a systematic review of randomized controlled trials
Source: BMC Pediatr. 2024 Feb 2;24:95. doi: 10.1186/s12887-024-04563-y (PMC10835972; doi:10.1186/s12887-024-04563-y)
Supplement: Supplementary file 1 — Additional file 1. [file 12887_2024_4563_MOESM1_ESM.doc]

APPENDIX

**SEARCH STRATEGY**

**PubMed**

((Back school OR back education programme OR back health OR postural education)) AND ((efficacy OR effectiveness OR importance))) AND ((children OR adolescents OR school-aged children))

**PEDro**

Effectiveness* Back school* children

Boolean operator-AND

**Google scholar**

Effectiveness of back care education programmes in school children back school OR back health OR postural education efficacy OR effectiveness OR importance children OR school-aged children OR adolescents back education programme

**Cochrane (CENTRAL)**

back school OR back education programme AND children AND adolescent AND efficacy

**HINARI**

((back school) OR (back education programme) OR (back health) OR (postural education)) AND (efficacy) OR (effectiveness) OR (importance)) AND ((children) OR (adolescents) OR school-aged children
